# Supplementary material for: Dynamical modeling of the H3K27 epigenetic landscape in mouse embryonic stem cells
Source: PLoS Comput Biol. 2022 Sep 2;18(9):e1010450. doi: 10.1371/journal.pcbi.1010450 (PMC9477427; doi:10.1371/journal.pcbi.1010450)
Supplement: S1 Table — Over all the tested cases, only r13 = r23 = 3 leads to a satisfying fit of the experimental profiles of H3K27 modifications around PcG-target genes (S3 Fig). Failure of the combination r13 = r23 = 4 is illustrated in S4 Fig. (DOCX) [file pcbi.1010450.s001.docx]

**S1 Table: Tested combinations of** $r_{13}$ **and** $r_{23}$**.** Over all the tested cases, only $r_{13}=r_{23}=3$ leads to a satisfying fit of the experimental profiles of H3K27 modifications around PcG-target genes (S3 Fig). Failure of the combination $r_{13}=r_{23}=4$ is illustrated in S4 Fig.

| $r_{13}$ | $r_{23}$ |
| --- | --- |
| 9 | 10,9,8 |
| 6 | 7,6,5 |
| 4 | 5,4,3 |
| 3 | 4,3,2 |
| 2 | 3,2,1 |
| 1 | 2,1,3 |
