# Supplementary figures and images for: Dynamical modeling of the H3K27 epigenetic landscape in mouse embryonic stem cells
Source: PLoS Comput Biol. 2022 Sep 2;18(9):e1010450. doi: 10.1371/journal.pcbi.1010450 (PMC9477427; doi:10.1371/journal.pcbi.1010450)

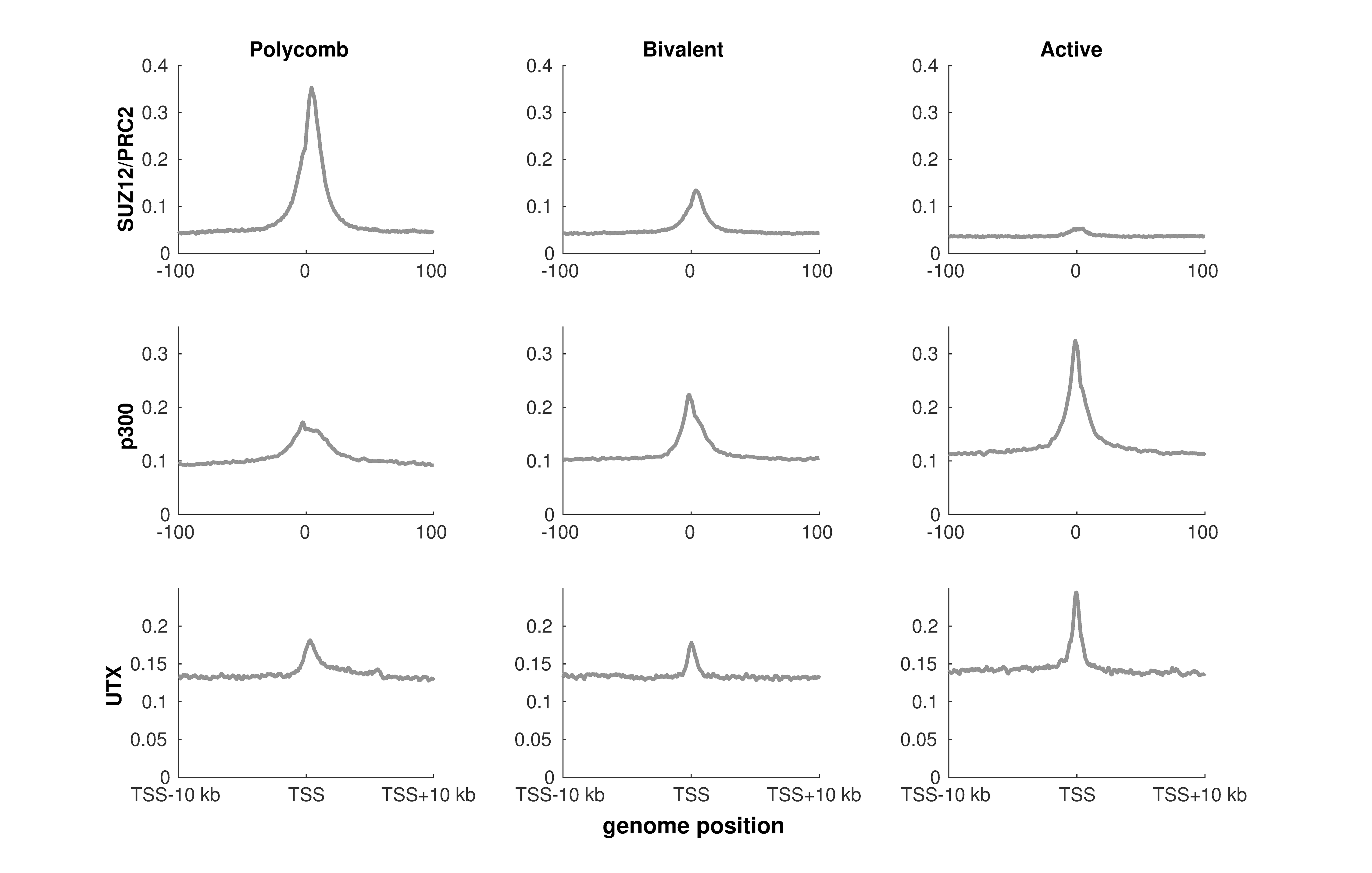

Supplement: S1 Fig — Average Chip-seq densities (normalized) of SUZ12, p300 and UTX of PcG-target, bivalent and active genes around the TSS in WT condition. (TIF) [file pcbi.1010450.s003.tif]

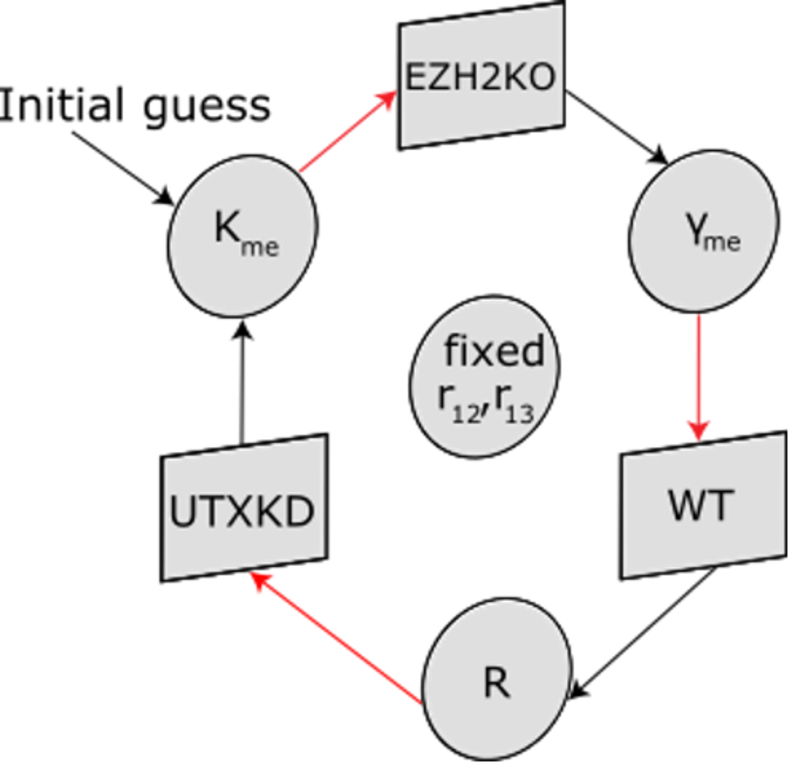

Supplement: S2 Fig — For fixed values of r12 and r23, an initial guess for kme is used to initialize an iterative inference cycle where a parameter inferred at one step feeds (red arrows) the next inference step based on various datasets (black arrows): EZH2KO data to infer γme, WT for R and UTXKD for kme (see main text). (TIF) [file pcbi.1010450.s004.tif]

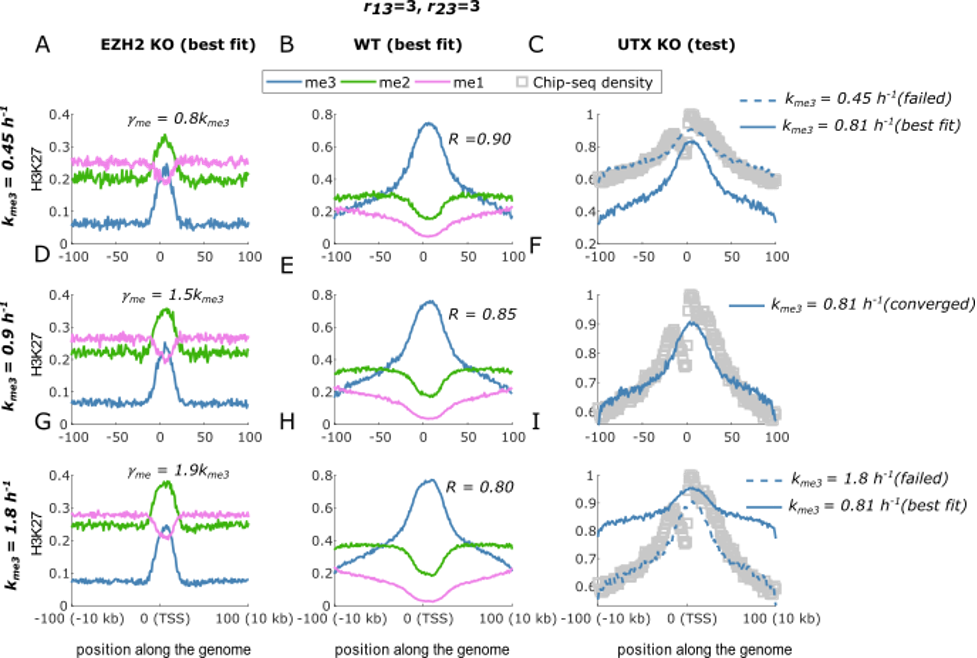

Supplement: S3 Fig — The steps for fixing R, kme3 and γme are illustrated. (A,D,G) H3K27 methylation are best fitted to EZH2 KO experimental profile by fixing γme for a particular kme3. (A,D,G) Then, H3K27 methylation are best fitted to WT experimental profile by fixing R for a fixed pair kme3, γme. (C,F,I) Finally the fixed parameters kme3, γme, R are tested if the H3K27me3 profile fits experimental density. (TIF) [file pcbi.1010450.s005.tif]

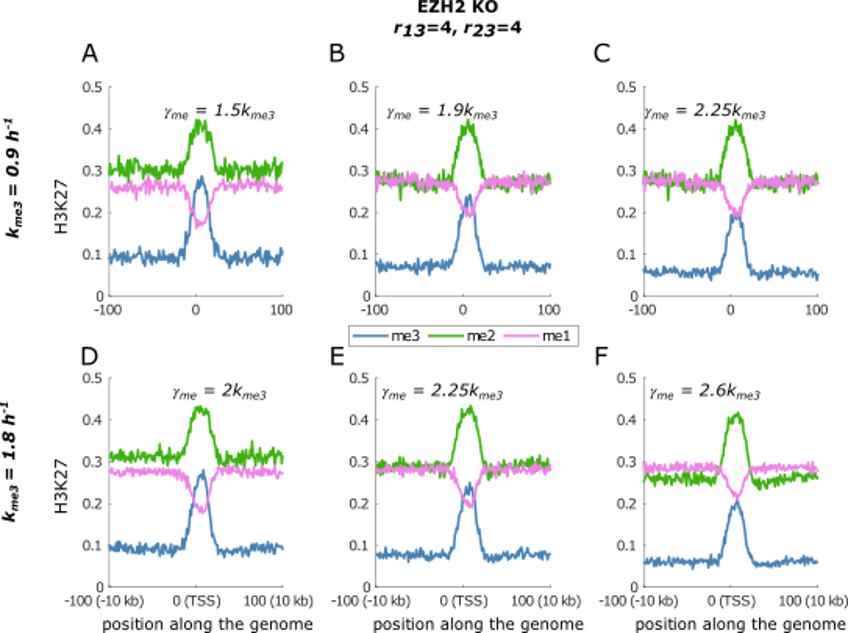

Supplement: S4 Fig — With these parameters, simulated H3K27 methylation profiles of EZH2 KO never capture the experimental methylation valency at promoters (Fig 2F of the main text). Top panel is for kme3 = 0.9 h−1 and explores γme to find a suitable kme3, γme pair to qualitatively capture methylation valency of EZH2KO. Bottom panel explores γme for kme3 = 1.8 h−1. (TIF) [file pcbi.1010450.s006.tif]

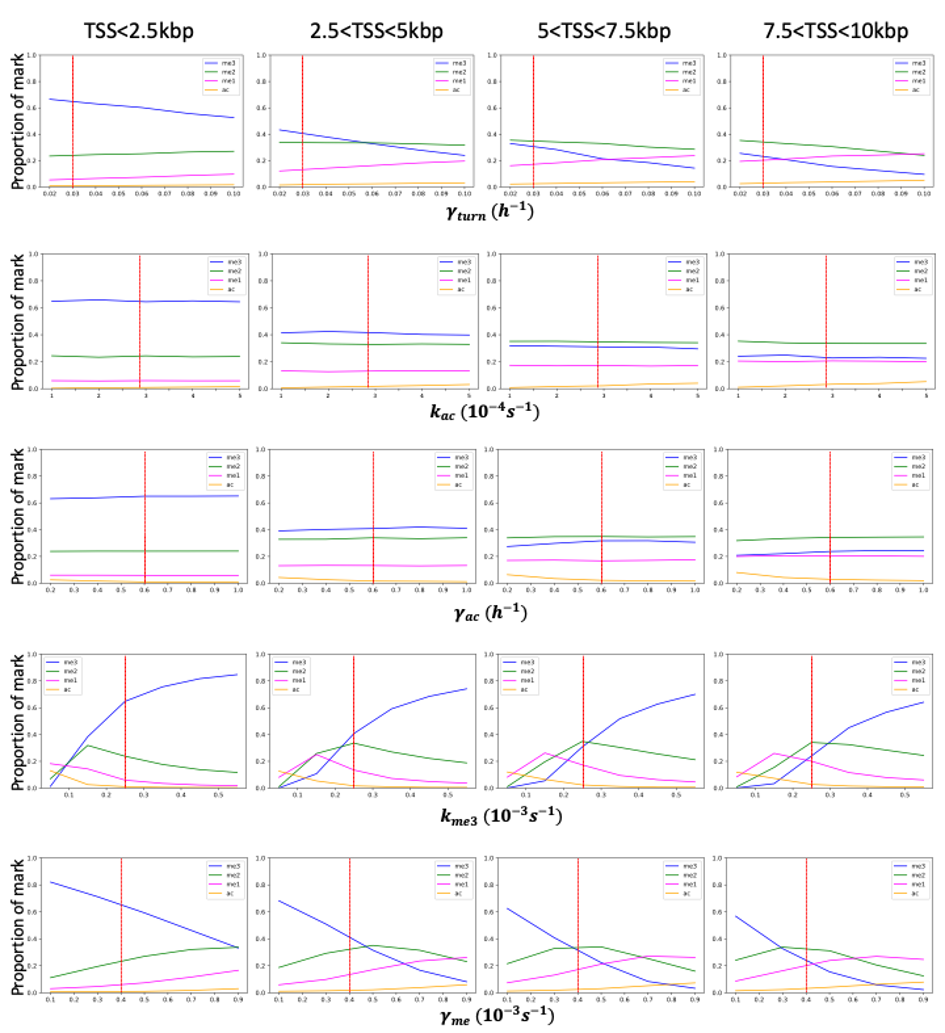

Supplement: S5 Fig — Average predicted proportion of a given mark around PcG-target genes as a function of the different model parameters, all other parameters fixed to WT values (red dotted lines). Panels from left to right correspond to regions close or far from TSS. For kme3, we also varied kme1 and kme2 by keeping r13 and r23 constant to WT values (see Table 1 of the main text). (TIF) [file pcbi.1010450.s007.tif]

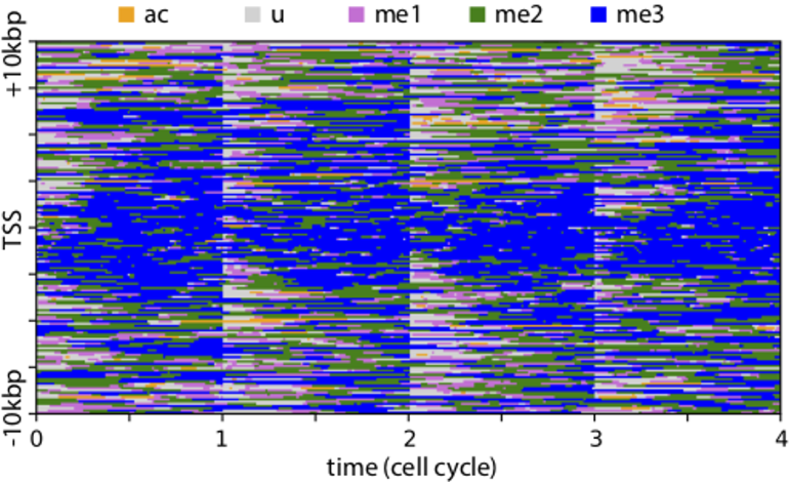

Supplement: S6 Fig — Kymograph representing a typical simulation trajectory at periodic steady-state around PcG-target genes with WT parameters obtained with the Gillespie algorithm. The local epigenetic state fluctuates stochastically following the kinetic rates given in the text. (TIF) [file pcbi.1010450.s008.tif]

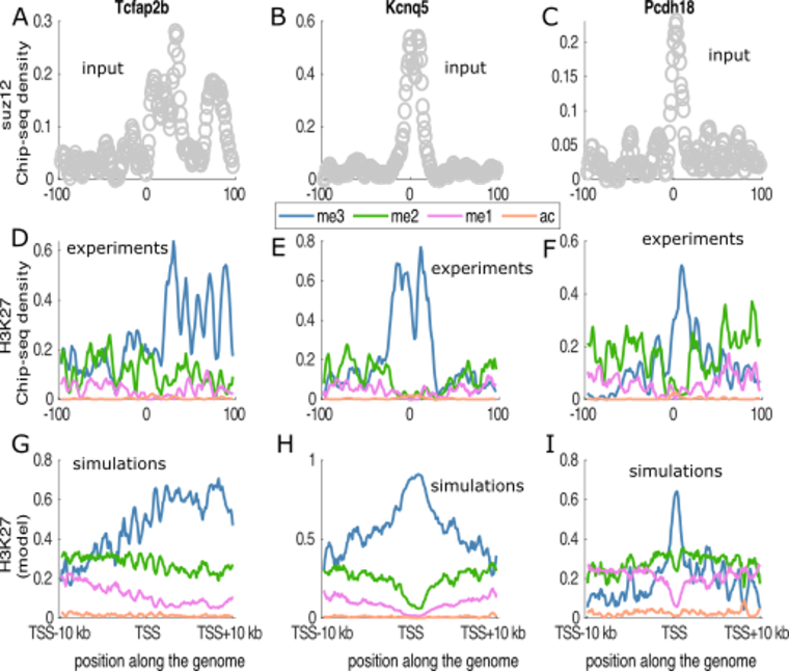

Supplement: S7 Fig — Predicting the H3K27 modification landscape at single genes in PcG-target domains. Profiles of gene Tcfap2b (left column), Kcnq5 (middle column), and Pcdh18 (right column). (First row) Input SUZ12 occupancies of three specific genes. (Second row) Chip-seq H3K27 methylation and acetylation for corresponding genes. (Third row) Simulated H3K27 modification profile of the respective genes. (TIF) [file pcbi.1010450.s009.tif]

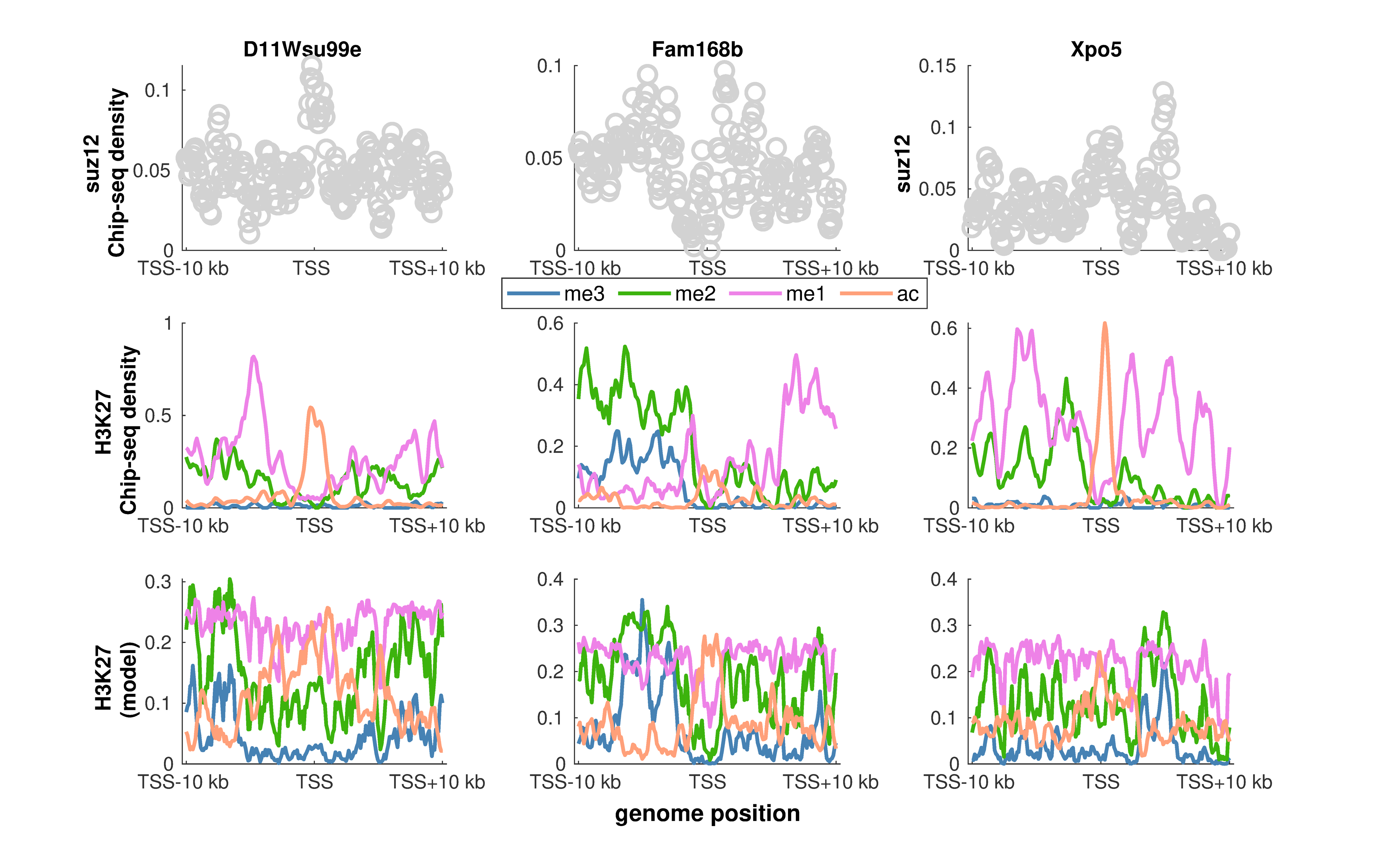

Supplement: S8 Fig — Predicting the H3K27 modification landscape at single genes of active domain. Profiles of gene D11Wsu99e (left), Fam168b (middle) and Xpo5 (right). (First row) Input SUZ12 occupancies of three specific genes. (Second row) Chip-seq H3K27 methylation and acetylation for corresponding genes. (Third row) Simulated H3K27 modification profile of the respective genes. (TIF) [file pcbi.1010450.s010.tif]

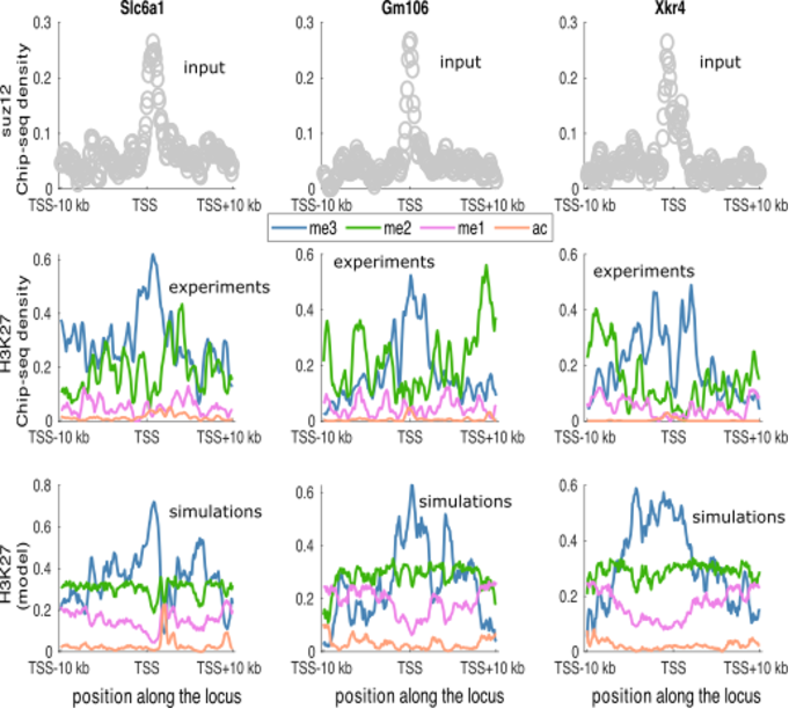

Supplement: S9 Fig — Predicting the H3K27 modification landscape at single genes of bivalent domain. Profiles of gene Scl6a1 (left), Gm106 (middle) and Xkr4 (right). (First row) Input SUZ12 occupancies of three specific genes. (Second row) Chip-seq H3K27 methylation and acetylation for corresponding genes. (Third row) Simulated H3K27 modification profile of the respective genes. (TIF) [file pcbi.1010450.s011.tif]

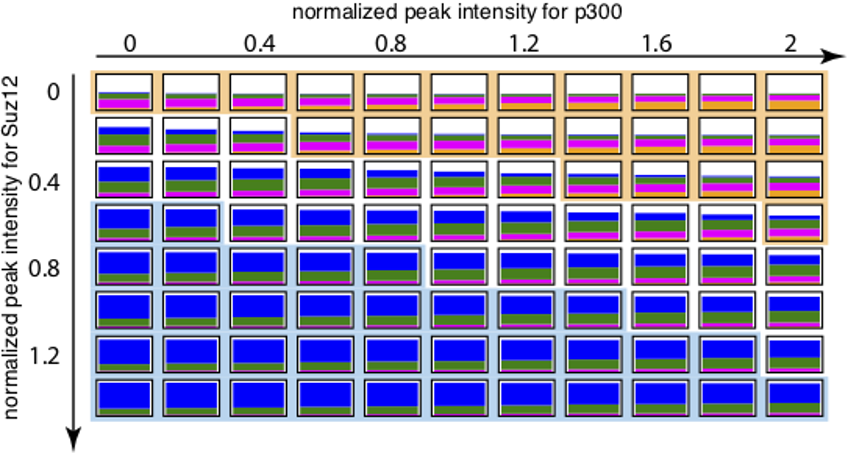

Supplement: S10 Fig — We varied the strengths of recruitment of p300 (x-axis) or Suz12/PRC2 (y-axis) around TSS for WT parameters. For each condition, we computed the average proportion of each H3K27 mark in a ±2.5kbp window around TSS. The corresponding stacked bar charts are given in the subplot (blue: me3, green: me2, magenta: me1, orange: ac, white: u). This allows us to define qualitatively two regions depending on the relative methylation valency: a PcG-target-like region (blue area) with me3>>me2>me1 and an active-like region (orange area) with me1>me2>>me3. (TIF) [file pcbi.1010450.s012.tif]

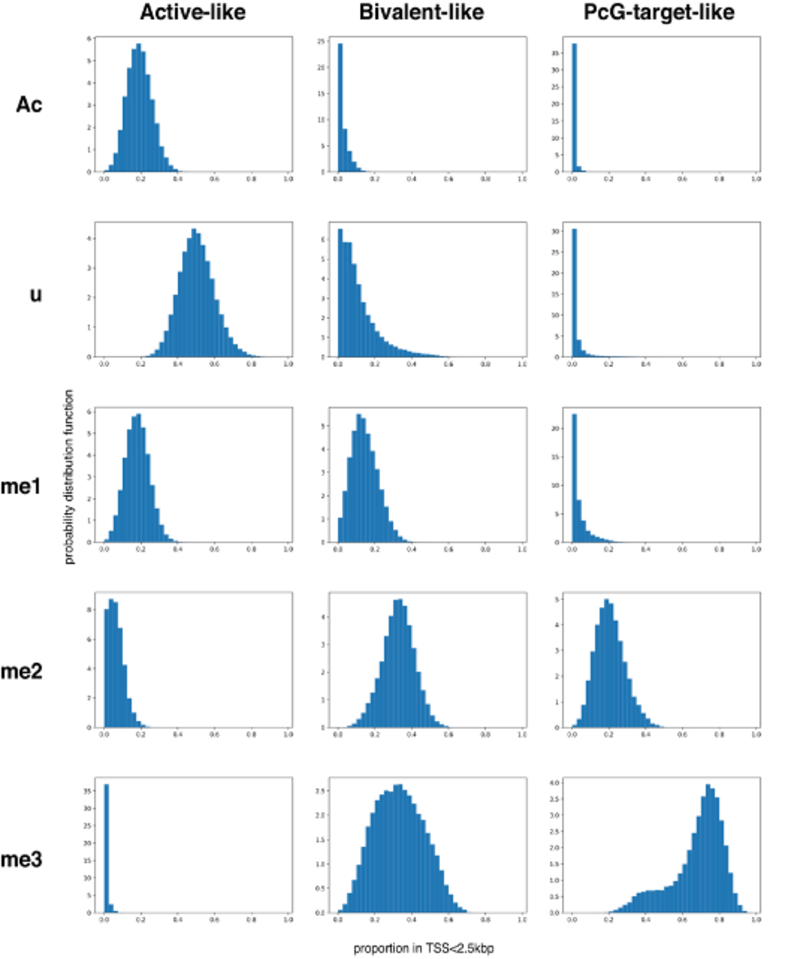

Supplement: S11 Fig — Probability distribution functions for the proportion of a H3K27 state inside the region TSS±2.5kbp in a population of unsynchronized cells for three values of HME recruitment strengths (Fig 5A of the main text), one in the Active-like region ((α;β)≈(0;1)), one with a bivalent-like inputs ((α;β)≈(0.4;0.5)) and one in the PcG-target-like region ((α;β)≈(1;0.3)). (TIF) [file pcbi.1010450.s013.tif]

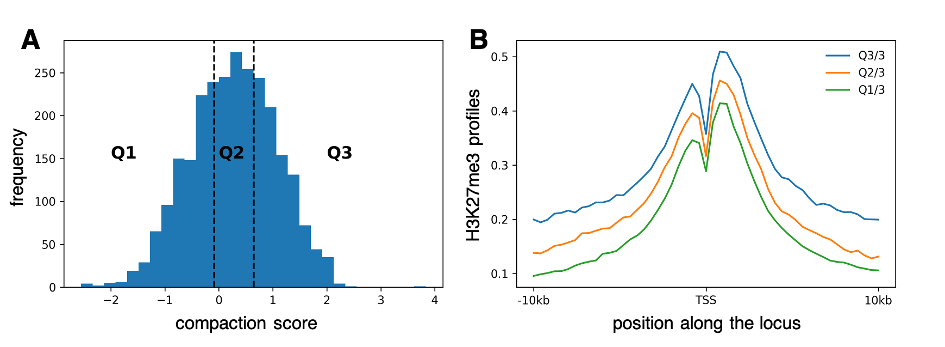

Supplement: S12 Fig — For each PcG-target gene, we estimated a compaction score that translates the density of 3D contacts around this gene. More precisely, we took the Hi-C data of mESCs at 10kbp resolution from (Bonev et al, Cell, 171: 557–572.e24, 2017) that we distance-normalized (the Hi-C value of each bin (i,j) is normalized by the average contact frequency at genomic distance |j-i|) to obtain the so-called observed-over-expected contact matrix OE. For a gene g with a TSS at position ig along the genome, we define its compaction score as the log2 of the median value of the OE matrix in a ±100kbp window around the TSS: log2[median{OE((ig−100kbp):(ig+100kbp);(ig−100kbp):(ig+100kbp))}]. The distribution of compaction scores in the ensemble of PcG-target genes is given in panel (A). We divided this ensemble into three subgroups of the same size: Q1 with low compaction scores, Q2 with intermediate and Q3 with high scores (A). Panel (B) shows the average H3K27me3 profiles around TSS for each subgroup (computed as the other average H3K27 profiles in the main text). The more compact the gene is the more extended the profile is. (TIF) [file pcbi.1010450.s014.tif]

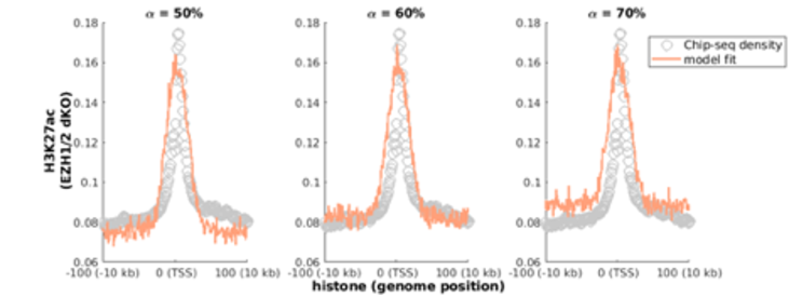

Supplement: S13 Fig — Experimental and fitted H3K27ac profile for different values of α. The best fit α = 0.6 is picked for which kac = 1.03 h−1. (TIF) [file pcbi.1010450.s015.tif]
