# Supplementary material for: Dynamical modeling of the H3K27 epigenetic landscape in mouse embryonic stem cells
Source: PLoS Comput Biol. 2022 Sep 2;18(9):e1010450. doi: 10.1371/journal.pcbi.1010450 (PMC9477427; doi:10.1371/journal.pcbi.1010450)
Supplement: Attachment — Submitted filename: Newar_et_al_comments_to_the_authors.pdf [file pcbi.1010450.s016.pdf]

Summary:

In this manuscript, Newar et al., based on previous biochemical information and available data sets, developed a mathematical model to predict a locus-specific distribution of H3K27 modifications in mouse embryonic stem cells (mESCs).

They focused on a 20kbp region centered on the transcription start sites of genes and took advantage of the various datasets available in several background in mouse ES cells with manipulated genotypes for both histone modifying enzymes. They designed a model including the known set of reactions governing the modification of H3K27, namely acetylation and methylation (mon, di, tri). For acetylation they considered the couple of the histone acetyltransferase and deacetylase, p300 and HDAC, and for methylation the methyltransferase and demethylase PRC2 and UTX. In calibrating the model, they involved the “reader”-“writer” capacity of PRC2. They conclude that the balance of H3K27ac/me modification is bimodal and highly fluctuating but in contrast with previous reports it is not bistable.

The choice of mESCs offers a lot of available datasets at TSS regions that they could exploit to calibrate the numerous parameters of the model. They first considered the TSSs of genes targeted by PRC2 (PcG-target genes) for the calibration. Then, they simulated the distribution of H3K27ac/me at “bivalent” and “active” genes, resulting in a qualitative match of the observations, notably in the close vicinity of the TSS. They next linked the distribution of the modifying enzymes (p300, and Suz12 for PRC2) with the relative presence of the various H3K27 modifications. They classified the genes in this way with their initial classification of “PcG-target”, “bivalent” and “active” genes. Finally, they explored the potential importance of histone renewal by turnover or through the replication process. They isolated in their measures the newly incorporated histones to evaluate the timing of their modifications along successive cell-cycles.

The authors provide a mathematical modelling of H3K27 modification dynamics by taking advantage of large sequencing data sets available in mESCs considering most of the known information on the topic. This is timely and potentially very interesting.

However, there are several limitations to fully apprehend this work, and several information are missing as well as clarifications

(1) While understanding how the simulations were run is accessible, the initial state of the nucleosome vector is not provided.

(2) Information concerning the definition of their gene classification is lacking, which would be expected at the beginning of the study. Fig.S8 may be of relevant in this classification but this is unclear. Fig.5 suggests no overlap between the three classes while it did not seem obvious.

(3) In addition, the effects of the genotype perturbations on how it can change the state of the gene are not analysed in sufficient depth since they can impair calibration of the parameters. For example, the Fig 2, EZH1/2 double knockout increased the expression level and may change the parameter Kac of wild type. This may be important to explain some of the discrepancies with their simulations on “bivalent” and “active” genes in addition to differences between gene classes.

(4) To better explore the “competition” between activating and repressing factors, the author could have simulated a change from “active” to “PcG-target” situation, or the inverse. In this way they could probe how the model work in a situation that could reflect differentiation.

(5) There is a need to evaluate how the model is affected when changing values of its various parameters. Except for the case of synchronized cells in their cycle, it is difficult to appreciate the importance of histone renewal on the final H3K27ac/me distribution. The same applies to acetylation by p300 at “PcG-target” genes (which does not seem to be very significative). This point would deserve further analysis or to stress the current limitations.

(6) The authors propose that the “reader”-“writer” capacity of PRC2 enhances H3K27me3 presence at the TSS but quickly discard the fact that the model does not fit with the data when moving 10kb away from the TSS. This aspect is important to consider when discussing the propagation aspect, that should build on this capacity while we would think it is due to this same capacity of PRC2.

(7) Finally, efforts should be made for the manuscript to be easier to read and follow: a serious editing is needed in particularly the figures and their references in the text are inconsistent.

In conclusions, while the current manuscript can bring a significant advance in modelling H3K27me dynamics at gene TSS in mESCs, it still needs improvements as listed above. This is critical to properly explain the methods leading to their gene classification with accurate details in the description of how the simulation works. More emphasis could be made on the importance of the parameters in the model behaviour. This would help to better understand some discrepancies between the simulations and the real data. We also trust that adding a few new simulations, in activation of a gene, could be achieved in a reasonable amount of time and would add significant value to this study. Should these points be considered carefully, along with improvement of the presentation, the paper could form a strong contribution of interest to Comp Biol Readership.

Detailed comments

- Details in figures are confusing. For example, Fig.4, top line is indicated with data from EZH1/2 DKO while half of the panels are from WT data. Several times references to Fig.4 are wrong mixing “active” and “bivalent” related panels.

- Definition of the gene classification should be provided upfront since the model calibration is performed on one of the classes: Description of the relation between the presence of histone modifying enzymes and the relative presence of the H3K27 modifications (current Fig.S8) should be provided before modelling. This will provide a description of the data that they will use along with the various existing patterns allowing them to define the “active” and “PcG-target” classes of genes. The “bivalent” class is not well supported. Thus, additional information is expected to justify it.

- The calibration strategy, notably for the PRC2 activity part, needs clarification. It would be a great help to provide a scheme describing the strategy of what they call a “converging loop” over the sequence of parameter optimization over the various datasets.

- Logic in the text and figures: as an example, Reference to Fig.1B before Fig.1A in the introduction part should be avoided. Also, mis-ordered usage of panels impairs the reading of the results, and it would be better to reorganize the concerned figures or how the results are described.

- Discussion on the effect of the various KO used to calibrate the model parameters would be useful.

- Additional comments to explain when the model does not fit with the data in particular away from the TSS and if this is related to the “reader”-“writer” capacity of PRC2, as it is even more intense when looking at individual genes.

- Variation in the simulated results according to variation in the parameter values should be provided. Notably, the turnover and replication in case of simulated asynchronous cells does not seem to be significant.

- In addition to the description of the link between presence of histone modifying enzymes and H3K27 modification profiles (current Fig.S8), simulations involving changes in histone modifying enzymes would help to appreciate how the model can account how to move from one profile to another.

- If PRC2 recruitment is mediated by H3K27me3, it is also regulated by other chromatin components, like H2AZ and H3.3 variants (Yan Wang et al. BMC Biol. 2018), this is currently not considered and would be worth to mention in the discussion.

- Since they discussed the fact that the 3D physical contact may result in long-range spreading parameter (R) increasing. To test this hypothesis, they should compare the inferred values for this parameter on “PcG-target” genes located in either “A” compartment or in “B” compartment.
