# Supplementary material for: Dynamical modeling of the H3K27 epigenetic landscape in mouse embryonic stem cells
Source: PLoS Comput Biol. 2022 Sep 2;18(9):e1010450. doi: 10.1371/journal.pcbi.1010450 (PMC9477427; doi:10.1371/journal.pcbi.1010450)
Supplement: Attachment — Submitted filename: Rebuttal+cover.pdf [file pcbi.1010450.s017.pdf]

Dear Editor,

Thank you for giving us the opportunity to submit a revised version of our manuscript entitled 'Dynamical modeling of the H3K27 epigenetic landscape in mouse embryonic stem cells' to *PLoS Computational Biology*. We greatly appreciate the time and efforts you and the reviewers have dedicated to providing valuable comments to our work. In particular, we are very enthusiastic about the very positive feedback provided by each reviewer. We have addressed all the concerns expressed by the reviewers and we firmly believe that our revised manuscript is now suitable for publication.

We would also like to apologize for the unexpected long time we took to make the revisions. Kapil Newar, the PhD student in charge of this work, had serious personal issues, and we had to organize ourselves to find a solution. Thus, 2 members of my lab (A.Z. Abdulla & H. Salari) helped us for the revision and are now co-authors of the study.

Briefly, our main revisions lie (1) in a more detailed description and discussion of the discrepancies between experiments and model predictions; (2) new simulations to make a systematic parameter sensitivity analysis and to investigate the kinetics of H3K27 profiles when silencing an active gene (and activating a silenced gene); (3) a deeper discussion of the limitations of the model; (4) a better description of methods used to simulate the model. Main changes are colored in red in the revised manuscript.

Please let us know if you need further information.

Best regards,

Daniel Jost on behalf of all co-authors (K. Newar, A.Z. Abdulla, H. Salari, E. Fanchon, D. Jost)

Below is a point-by-point response to reviewers concerns.

Comments from the reviewers are shown in Courier font.

Responses to the comments are shown in blue.

Reviewer #1

Summary:
In this manuscript, Newar et al., based on previous biochemical information and available data sets, developed a mathematical model to predict a locus-specific distribution of H3K27 modifications in mouse embryonic stem cells (mESCs). They focused on a 20kbp region centered on the transcription start sites of genes and took advantage of the various datasets available in several background in mouse ES cells with manipulated genotypes for both histone modifying enzymes. They designed a model including the known set of reactions governing the modification of H3K27, namely acetylation and methylation (mon, di, tri). For acetylation they considered the couple of the histone acetyltransferase and deacetylase, p300 and HDAC, and for methylation the methyltransferase and demethylase PRC2 and UTX. In calibrating the model, they involved the "reader"-"writer" capacity of PRC2. They conclude that the balance of H3K27ac/me modification is bimodal and highly fluctuating but in contrast with previous reports it is not bistable. The choice of mESCs offers a lot of available datasets at TSS regions that they could exploit to calibrate the numerous parameters of the model. They first considered the TSSs of genes targeted by PRC2 (PcG-target genes) for the calibration. Then, they simulated the distribution of H3K27ac/me at "bivalent" and "active" genes, resulting in a qualitative match of the observations, notably in the close vicinity of the TSS. They next linked the distribution of the modifying enzymes (p300, and Suz12 for PRC2) with the relative presence of the various H3K27 modifications. They classified the genes in this way with their initial classification of "PcG-target", "bivalent" and "active" genes. Finally, they explored the potential importance of histone renewal by turnover or through the replication process. They isolated in their measures the newly incorporated histones to evaluate the timing of their modifications along successive cell-cycles. The authors provide a mathematical modelling of H3K27 modification dynamics by taking advantage of large sequencing data sets available in mESCs considering most of the known information on the topic. This is timely and potentially very interesting.

We thank Reviewer 1 for his/her overall positive assessment of our work and his/her insightful remarks and suggestions that help us improve the manuscript.

However, there are several limitations to fully apprehend this work, and several information are missing as well as clarifications
(1) While understanding how the simulations were run is accessible, the initial state of the nucleosome vector is not provided.

Our simulations always start in a fully unmodified state (all histones with a 'u' state). Moreover, they were run long enough to reach a periodic steady-state, independent of the initial configuration, before taking any measurements or making any perturbations. We added this information in the subsection 'Simulations of the model' of the 'Materials and Methods' (Page 26).

(2) Information concerning the definition of their gene classification is lacking, which would be expected at the beginning of the study. Fig.S8 may be

of relevant in this classification but this is unclear. Fig.5 suggests no overlap between the three classes while it did not seem obvious.

The gene classification we used was obtained from Chory et al (Ref 25), in which authors took several histone marks including H3K27 modifications but also RNAseq data to classify genes into 5 categories (highly active, lowly active, bivalent, repressed and background) in mESC. In relation with H3K27 modifications, we decided to focus on highly active, bivalent and repressed genes. For each list of genes, we then computed the experimental average profiles of HMEs and H3K27 histone marks around their TSS. Looking at the experimental histone profiles, these three gene categories exhibit in average a different "valency of H3K27 methylation", defined as the relative levels of mono-, bi- and trimethylation in a +/- 2.5 kbp region around TSS. me3>>me2>me1 for PcG-target-genes, me1>me2>>me3 for active genes, me2~me3>me1 for bivalent genes. Since our model only predicts H3K27 modifications (and not all the other information used by Chory et al to categorize genes), in our systematic analysis of the differential recruitment of HMEs (Fig.5 & Fig.S10), we used such qualitative valency criterion to define PcG-like or active-like profiles. We insist on the '-like' term as our model cannot predict if the genes will be actually silenced or transcribed. We also removed reference to the bivalent-like class as it is not well supported and have preferred the term 'intermediate' situations to talk about valencies that are not active-like or PcG-like.

In the revised version of the manuscript, we clarify this point by (i) adding a paragraph at the end of section 'A dynamical model for the regulation of H3K27 epigenetic modifications in mESC' (Page 7) to define the gene categories and the notion of valency (definitions that were less detailed and buried in the introduction in the first version of the manuscript); (ii) rephrasing the definition of the qualitative "like" behaviors in the subsection 'Competition between activating and repressing factors shapes the local epigenomic landscape' (Page 15); (iii) adding a brief description of the inference of gene categories made by Chory et al in the subsection 'Chip-seq data and analysis' of the 'Materials and methods' (Pages 23-24); (iv) adding a more detailed description of how we build the 'phase diagram' in Fig.5A based on the valencies (new Fig.S10) in a new subsection 'Phase diagram driven by differential HME recruitment' in the 'Materials and Methods' (Page 29-30). Moreover, we believe also that the new version of Fig.1 also facilitates the understanding of this point.

(3) In addition, the effects of the genotype perturbations on how it can change the state of the gene are not analysed in sufficient depth since they can impair calibration of the parameters. For example, the Fig 2, EZH1/2 double knockout increased the expression level and may change the parameter Kac of wild type. This may be important to explain some of the discrepancies with their simulations on "bivalent" and "active" genes in addition to differences between gene classes.

Indeed, in our inference scheme, we made the simplification that, in the different mutant strains, except the corresponding 'mutated' parameters that were set to zero, all the other parameters were not perturbed. We now added a paragraph in the Discussion, discussing that approximation (Page 22).

(4) To better explore the "competition" between activating and repressing factors, the author could have simulated a change from "active" to "PcG-target" situation, or the inverse. In this way they could probe how the model work in a situation that could reflect differentiation.

We thank the reviewer for this interesting suggestion. We add two panels in Fig.5C tracking the dynamics of H3K27 modifications when switching from PcG-target-like parameters to active-like parameters and vice versa. We observed dynamics that are consistent with our previous analysis (Fig.6). For example, from active-like to PcG-target-like HME profiles (Fig.5C top), the situation is rather similar to Fig.6G-H where we were studying the reestablishment of the epigenetic landscape after inhibition of PRC2. Close to the

TSS, establishment is faster (~ one cell cycle *T*) as mostly driven by the on-site action of HMEs. However at longer-range, establishment is slower (~ 2*T*) as there is a delay (~*T/2*) corresponding to the time needed for the region close to the TSS to be trimethylated enough to allow allosteric long-range spreading. We add a paragraph at the end of the section 'The model captures the maintenance and spreading dynamics of H3K27me3' to discuss that (Page 18).

(5) There is a need to evaluate how the model is affected when changing values of its various parameters. Except for the case of synchronized cells in their cycle, it is difficult to appreciate the importance of histone renewal on the final H3K27ac/me distribution. The same applies to acetylation by p300 at "PcG-target" genes (which does not seem to be very significant). This point would deserve further analysis or to stress the current limitations.

As suggested by the reviewer, we now performed a more systematic parameter analysis for PcG-target genes along the same line as we already did for the R-parameter in the first version of the manuscript (Fig.3A). We added a panel in Fig.3 (Fig.3B), a Supplementary figure (Fig.S5) and a paragraph in the section 'PRC2 spreading efficiency dictates the shapes, valencies and correlations of methylation profiles' (Page 11) to discuss that. In particular, cell cycle length does have a significant effect at low values on the profiles by favoring the u state and thus limiting the allosteric effect, as observed and discussed already in previous modeling works (Zerihun et al, Phys Biol 2016 ; Reinig et al, Nat Com 2020).

(6) The authors propose that the "reader"-"writer" capacity of PRC2 enhances H3K27me3 presence at the TSS but quickly discard the fact that the model does not fit with the data when moving 10kb away from the TSS. This aspect is important to consider when discussing the propagation aspect, that should build on this capacity while we would think it is due to this same capacity of PRC2.

We now added a paragraph in the section 'Inference of methylation-related rates using EZH2 KO, WT and UTX KD profiles' (Pages 10-11) to describe and discuss the discrepancies between predictions and experiments for PcG-target genes, including the observation that some profiles (for example the slowly-decreasing profile of H3K27me2 in EZH2 KO cells) are not well captured by the model.

(7) Finally, efforts should be made for the manuscript to be easier to read and follow: a serious editing is needed, in particular the figures and their references in the text are inconsistent.

We performed a careful proofreading and corrected the misassigned references to figure panels.

In conclusion, while the current manuscript can bring a significant advance in modelling H3K27me dynamics at gene TSS in mESCs, it still needs improvements as listed above. This is critical to properly explain the methods leading to their gene classification with accurate details in the description of how the simulation works. More emphasis could be made on the importance of the parameters in the model behaviour. This would help to better understand some discrepancies between the simulations and the real data. We also trust that adding a few new simulations, in activation of a gene, could be achieved in a reasonable amount of time and would add significant value to this study. Should these points be considered carefully, along with improvement of the presentation, the paper could form a strong contribution of interest to Comp Biol Readership.

Detailed comments

- (8) Details in figures are confusing. For example, Fig.4, top line is indicated with data from EZH1/2 DKO while half of the panels are from WT data. Several

times, references to Fig.4 are wrong, mixing "active" and "bivalent" related panels.

We are sorry for the confusion. We slightly modified Fig.2 & 4 to avoid that.

- (9) Definition of the gene classification should be provided upfront since the model calibration is performed on one of the classes: Description of the relation between the presence of histone modifying enzymes and the relative presence of the H3K27 modifications (current Fig.S8) should be provided before modeling. This will provide a description of the data that they will use along with the various existing patterns allowing them to define the "active" and "PcG-target" classes of genes. The "bivalent" class is not well supported. Thus, additional information is expected to justify it.

See point 2 above.

- (10) The calibration strategy, notably for the PRC2 activity part, needs clarification. It would be a great help to provide a scheme describing the strategy of what they call a "converging loop" over the sequence of parameter optimization over the various datasets.

We add a scheme in the new Fig.S2 to describe the iterative inference strategy.

- (11) Logic in the text and figures: as an example, Reference to Fig.1B before Fig.1A in the introduction part should be avoided. Also, mis-ordered usage of panels impairs the reading of the results, and it would be better to reorganize the concerned figures or how the results are described.

In the revised manuscript, we tried to avoid such mis-ordered usage (see also point 7 above). However, the first reference to Fig.5C still appears after the first one to Fig.6 as we believed it facilitates the comprehension of the figures while not perturbing the reading of the text.

- (12) Discussion on the effect of the various KO used to calibrate the model parameters would be useful.

See point 3 above.

- (13) Additional comments to explain when the model does not fit with the data in particular away from the TSS and if this is related to the "reader"-"writer" capacity of PRC2, as it is even more intense when looking at individual genes.

See point 6 above.

- (14) Variation in the simulated results according to variation in the parameter values should be provided. Notably, the turnover and replication in case of simulated asynchronous cells does not seem to be significant.

See point 5 above.

- (15) In addition to the description of the link between presence of histone modifying enzymes and H3K27 modification profiles (current Fig.S8), simulations involving changes in histone modifying enzymes would help to appreciate how the model can account how to move from one profile to another.

See point 4 above.

- (16) If PRC2 recruitment is mediated by H3K27me3, it is also regulated by other chromatin components, like H2AZ and H3.3 variants (Yan Wang et al. BMC Biol. 2018), this is currently not considered and would be worth to mention in the discussion.

We thank the reviewer for this suggestion. We now have mentioned that in the Discussion (Page 22).

- (17) Since they discussed the fact that the 3D physical contact may result in long-range spreading parameter (R) increasing. To test this hypothesis, they should compare the inferred values for this parameter on "PcG-target" genes located in either "A" compartment or in "B" compartment.

We thank the reviewer for this suggestion. However, the detailed analysis of the coupling between 3D organization and epigenetic landscape is beyond the scope of this article and will require extended work that will be performed in a future, dedicated study. Nevertheless, to illustrate the relation between 3D compaction and long-range spreading, we computed the average H3K27me3 ChIPseq profiles around PcG-target genes, embedded in more or less compact regions (Fig.S12). We clearly observed that genes in compact regions have more extended profiles. We added a reference to this supplementary figure in the Discussion (Pages 22-23).

Reviewer #2:

This paper deals with an issue of current interest, namely, the mechanisms underlying the formation and sustenance of patterns of chromatin modifications or epigenetic landscape. Such landscapes are essential to the maintenance of cell identity and are known to be deteriorated in a number of situations, for example, aging. Their model is based on a data-driven component, where the distribution of several epigenetic enzymes are extracted from Chip-seq data, and a modelling component, where several mechanisms for chromatin modifications are explored. Specifically, the authors provide an interesting exploration of the role of allostery in the PRC2 complex, which allows for non-local interactions between modified chromatin residues (H3K27me3). The methodology is sound and their results are interesting and in agreement with current experimental knowledge, so that I recommend this paper to be accepted for publication.

We thank the reviewer for his/her enthusiasm. His/her remarks really help us to improve the quality of our work.

However, I have some comments regarding issues that I think are in need of clarification.

1.- In Eq. (1), Description of the stochastic epigenetic model, the non-local part of the methylation rate is assumed to decrease as a power of the distance between loci measured in base pairs. Whilst this is a reasonable assumption, it is unclear whether it faithfully reflects the 3D structure of DNA which ultimately gives rise to the contacts between distant loci. Although later on the authors provide a rationale for this model in terms of polymer-physics argument, the authors should further clarify their assumption (for readers unfamiliar with polymer physics) and also comment on how the model could be modified in order to account for a network of contacts extracted from data.

In Eq.1, the second term of its right part corresponds to the long-range spreading part. In the revised version, we reformulated it into a more general expression accounting for the probability $P_{3D}(i,j)$ that two loci i and j are in contact by DNA looping (Page 25). In our work, to simplify the analysis and also because we are mainly modeling *average* profiles around genes, we assumed that $P_{3D}(i,j) \sim 1/|i-j|^{\lambda}$ only depends on the genomic distance $|i-j|$ between i and j. Indeed, analysis of many experimental Hi-C data showed that, in average, $P_{3D}(i,j) \sim 1/|i-j|^{\lambda}$, with $\lambda \approx 1$ in mESC (Bonev et al, Cell 2017). Moreover, such type of scaling laws for $P_{3D}(i,j)$ is characteristic of the polymeric nature of chromatin (Mirny, Chromosome Res 2011). We now clarified this point in the subsection 'Mathematical formulation of the kinetic transition rates' in Materials and Methods (Page 25).
However, a priori, the model can be run for any $P_{i,j}$ matrix to account for locus-dependent networks of contacts. We added a sentence to discuss that in the Discussion section (Page 22).

2.- Another issue regarding model formulation is that the authors consider only (positive) feedbacks between methylation marks. Other authors have considered other feedbacks between modifications and enzyme activity. Could the authors clarify this point?

Indeed, other modeling works of epigenetic regulation have also integrated other feedback loops. For example: (1) in the original three-state generic model, originally developed by Dodd & Sneppen, 3 more feedbacks are considered: the methylation/inactive state promotes the deacetylation of the active state; the acetylation/active state promotes the demethylation of the inactive state and the acetylation of the

unmodified state ; (2) in recent modeling works by Howard’s group on PcG regulation (Berry et al, 2017 and Holoch et al, 2021), in addition to the positive feedback on methylation (as in our model), another feedback was integrated: transcription promotes demethylation (eg, via an increase in histone turnover or via the recruitment of demethylase), and methylation inhibits transcription.

In our model of PcG regulation in mESC, we only considered the positive feedback loops between methylation marks for several reasons: (i) we focused on well-established mechanisms and there is no clear evidence for the recruitment of HDAC by H3K27me3-marked histones or the promotion of acetylation by acetylated state at promoter (already discussed in the text in subsection ‘Addition and removal of H3K27 acetylation’, page 6) ; (ii) we mainly focused our work on PcG-target genes for which the feedbacks mediated by acetylation/active/transcribed state are likely to be weak. For example, in mESCs, since histone turnover rate may vary by at most ~2 fold (Chory et al, 2017) and H3K27 profiles are only weakly dependent on histone turnover around PcG-target gene (first row in new Fig.S5), we do not expect a state-dependent histone turnover (higher turnover for ac state) would significantly impact the profiles. Moreover, the effect of transcription/active states on the demethylation dynamics is already integrated in our model but as an open loop (not a closed feedback loop as in Howard’s models) since the profile of the demethylase UTX is an input of the model and is highly correlated to p300 occupancy and the transcriptional status.
We added a paragraph in the Discussion section to discuss that point (Page 21).

Reviewer #3:

The paper suggests a model for deducing relative strength of methylation reaction associated to PRC2 by analyzing how acetylation and me1, me2 and me3 marks are spatially distributed around a promoter. The overall assumption is that the output modification state is a direct function of the input state, and thus that there is no significant feedback for histones in me3 states recruiting PRC2, which again makes new me3.

Overall the work presents an interesting approach: By comparing experiments with and without long range methylations from the assumed PRC2 binding sites the authors identify the me2-->me3 state as rate limiting. Also the overall estimates of rates seem reasonable.

We thank Reviewer 3 for his/her overall positive assessment of our work and his/her insightful remarks and suggestions that help us improve the manuscript.

Main objection is the moderate quality of the fit to data.

We agree with Reviewer 3 that the fit of the data is not fully quantitative. However, we would like to note that our work is, to our knowledge, one of the first attempts (with Chory et al, 2019) that aims at describing profiles of H3K27 modifications around TSS using a rather simple predictive model. In the Discussion section, we discuss the limitations of our model and suggest future improvements.

(1) First of all, when reading figures, can the relative levels of different modifications be compared in experiments? and do their absolute levels mean anything?

The reviewer raises an important technical point that was addressed in the first version of the manuscript but was not clear enough and was buried into the subsection ‘Chip-seq data analysis’ in Materials and Methods. In the revised manuscript, we decided to dedicate a paragraph about that point at the beginning of the subsection ‘Parameter inference’ of Materials and Methods (Pages 27-28). Briefly, (1) we used a normalization method of quantitative ChIP-seq data that was developed by Chory et al (Mol Cell 2019) to precisely perform relative inter-mark comparisons ; (2) we fitted average profiles (i.e. average over many genes) that may smooth out any sequence-specific biases that may arise in ChIP experiments; (3) for most of the ChIP data we used, we make it clear that absolute levels cannot simply be interpreted as absolute probabilities but rather as being proportional to probabilities. That is why we are mostly performing qualitative fit on the methylation valencies. The only exception is the MINUTE ChIP data for UTXKD condition that, after normalization, was shown to be representative of an absolute probability (Kumar et al, Cell Rep, 2019).

(2) Fig. 2 F: The model have constant levels of modifications outside TSS, while the experiments for me2,me3 show longer range dependencies of distances (in fact this failure of reproducing large distance trends go through many of the figures,)
(3) Fig. 2 I: levels boosted by factor 2 in model while y-axis in experiment unchanged. Please comment on this and I would advise to be modest in claims (is long range recruitment too strong in model?)

(4) Overall distance dependence of ac state appears to be opposite in model and experiment.

We agree with the reviewer. We now have added a paragraph at the end of the section ‘Inference of methylation-related rates using EZH2 KO, WT and UTX KD profiles’ (Pages 10-11) where we described

and discussed the discrepancies observed in Fig.2 between experiments and model predictions, including the slowly-decreasing shape of H3K27me2 profile in EZH2 KO cells, the slight increase in H3K27ac around the TSS in EZH2 KO and WT cells, and the 'boost' factor for the methylation profiles in WT cells.

(5) Fig. 4 F does not resemble panel E: The me1 in model are much more localized. ac levels in experiments are 4 fold enhanced at TSS while model only have 2 fold. Other panels in Fig. 4 show similar discrepancies, in various directions. Overall the above does not inspire confidence for the reader, so please clarify.

We agree with the reviewer. Such descriptions of the many discrepancies were already present in the first version of the manuscript. In the revised one, we discuss in more detail the limits of the model, in particular regarding our description of active and bivalent categories and possible missing ingredients that may improve the modeling for these gene categories (see the new Discussion).

(6) From the pedagogical side, it is first quite late in the manuscript that one understands that everything is just a readout of an assumed PCR2, profile. It is mentioned early, but only briefly and Fig. 1B only gives a qualitative picture of something added. How Much is added and where? Can P300 and UTX at all change anything away from the TSS (since they seem to be a delta function there). How wide is the PRC2 profile added? (presumably that directly sets the width of the me2,me3 peak around TSS) Overall Fig 1 does a poor job in explaining the model. What do one really learn from panel A in that figure?

In the Introduction (Page 3, "the recruitment of HMEs at specific sites and their local and long-range spreading activities from these sites are designing the epigenetic H3K27 landscape in mESCs.") and in the first subsection of the Results part (Page 4, "we consider that the *de novo* establishment and maintenance of H3K27 modifications in mESCs is mainly carried out by the stable recruitment of HMEs to their cognate DNA recruitment sites and that the patterns of epigenetic marks around genes result from a complex network of local and long-range spreading or erasing mechanisms mediated by these HMEs" ; and Page 7 "This epigenetic model takes as inputs the binding profiles of HMEs like PRC2, p300 or UTX (Figs.1B and S5) and, for a given set of (de)methylation and (de)acetylation rates (Table 1), makes predictions about the corresponding profiles of H3K27ac/u/me1/me2/me3 modifications based on the simulations of many single-cell stochastic trajectories of small regions of the epigenome (see Materials and Methods). "), we already mentioned that indeed H3K27 profiles are readouts of HME binding profiles in the first version of the manuscript.

In addition, we have now reshaped Figure 1(and its caption) to clarify this point by adding realistic profiles of HMEs for the different gene categories and by redrawing panel A to better describe the model, in particular the long-range allosteric spreading.
